# Transportation protocols for accurate assessment of microbial burden classification using molecular methods

**DOI:** 10.1038/s41598-021-95619-x

**Published:** 2021-08-09

**Authors:** Amelia Kung, Jade Chen, Michael Tomasek, Dakai Liu, William Rodgers, Vincent Gau

**Affiliations:** 1 GeneFluidics, Los Angeles, CA USA; 2 Department of Pathology and Clinical Laboratories, New York-Presbyterian Queens, Flushing, NY USA

**Keywords:** Biotechnology, Microbiology, Molecular biology, Medical research

## Abstract

Point-of-care testing is cost-effective, rapid, and could assist in avoiding hospital visits during a pandemic. However, they present some significant risks that current technologies cannot fully address. Skin flora contamination and insufficient specimen volume are two major limitations preventing self-collection microbiological testing outside of hospital settings. We are developing a hybrid testing procedure to bridge the laboratory test with patient-side specimen collection and transportation for molecular microbial classification of causative bacterial infection and early identification of microbial susceptibility profiles directly from whole blood or urine specimens collected patient-side by health care workers such as phlebotomists in nursing homes or family clinics. This feasibility study presents our initial development efforts, in which we tested various transportation conditions (tubes, temperature, duration) for direct-from-specimen viable pathogen detection to determine the ideal conditions that allowed for differentiation between contaminant and causative bacteria in urine specimens and optimal growth for low-concentration blood specimens after transportation. For direct-from-urine assays, the viable pathogen at the clinical cutoff of 10^5^ CFU/mL was detected after transportation with molecular assays while contaminants (≤ 10^4^ CFU/mL) were not. For direct-from-blood assays, contrived blood samples as low as 0.8 CFU/mL were reported positive after transportation without the need for blood culture.

## Introduction

The clinical interpretation and cost-effectiveness of urine and blood cultures depend on many variables, the most important of which is the proportion of cultures that are contaminated by skin flora^1,2^. It is of paramount importance for specimens to be collected in such a way that contamination by indigenous flora is minimized. This is especially true for cultures of blood and urine or fluids in which infection is often caused by indigenous flora and for specimens collected from sites of putative infection that are contiguous to, or immediately adjacent to, cutaneous or mucosal surfaces^3^. Contaminations are most often attributed to the transfer of microorganisms from the immediate environment of the patient, skin microbiota, or, more rarely, from healthcare workers' hands^2,4^. Risks of exposure to such contaminations cannot be tolerated as the predictive value of urine and blood culture time to positivity is based on the premise that the bacterial inoculum in a true bacteremia is higher and grows faster than in a urine and blood culture contaminant’s^1^. This risk of contamination may be mitigated by the assistance of a healthcare provider. Furthermore, when a coagulase-negative staphylococci is isolated, a longer growth time is usually considered in favor of a contaminant, and growth times between contaminants and true pathogens overlap^5,6^.

The specimens for microbiological testing must also be collected with use of strict aseptic technique from anatomic sites most likely to yield pathogenic microorganisms, therefore bacterial cultures are not presently ready for home tests with self-collected specimens. The blood volume needed for blood culture and the need of a phlebotomist also exclude the use of finger prick blood samples. Sufficient material must be submitted for cultures and other tests, and blood volume is crucial to ensure accuracy in blood cultures. With limitations on self-collection methods due to the required volume for blood cultures and concerns regarding contamination by skin flora for both urine and blood cultures, the presence of healthcare professionals at collection sites is essential to ensure accurate clinical interpretation of culture results. Thus, point-of-care home tests with self-collected urine or blood samples are currently not feasible. There is a need for better interim tests between clinical laboratory cultures in hospital settings and self-collection home tests to rapidly identify patients with resistant pathogens and for more judicious use of broad-spectrum antibiotics for empiric sepsis treatment, especially in out-of-hospital settings. The specimen collection and transportation protocols developed in this study aimed to utilize the specimen transportation time as part of the viability preservation for urine cultures and viability enhancement for blood cultures. The impact of the different transportation conditions on assay performance was prioritized on differentiating contaminants from causative pathogen for urine specimens and low limit of detection for blood specimens. The assay sensitivity (true Positive rate) and specificity (true Negative rate) were not verified in this study and must be included in future clinical validation studies in which additional specimens are collected with consent for parallel testing with the gold standard blood/urine culture to identify contaminants based on the current clinical practice, such as blood culture bottles that turn positive in 2/3 sets collected.

**Figure 1:**
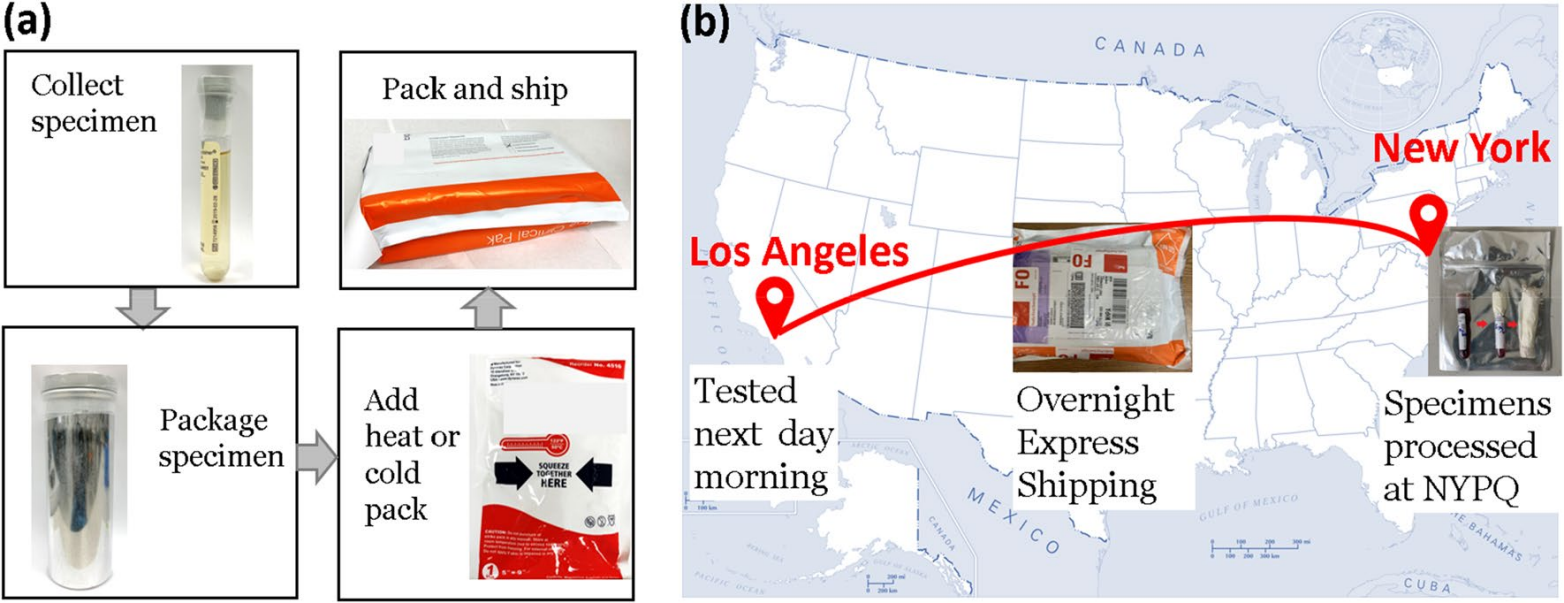
(**a**) Patient-side specimen collection and transportation package, (**b**) specimens shipped from NYPQ via FedEx Clinical Pak. Map was generated by a search on Wikimedia with "no-restriction" license rights (https://commons.wikimedia.org/wiki/File:US_state_outline_map.png). We then recolored it in Microsoft PowerPoint and added the locations of Los Angeles and New York.

A recent study demonstrated that the measurement of 21 blood biomarkers from 134 blood samples taken by emergency medical services (EMS) and placed in a Coleman cooler box on the worktop inside the patient’s cabin in an ambulance truck during the hospital transfer was exactly like the one immediately analyzed in the hospital setting^7^. The possible benefits to patient outcome deriving from out-of-hospital blood sampling were limited in this study, because blood samples were taken by EMS, and the time saved is only 30 min or less on the ambulance ride to the emergency department in the hospital. However, in the context of nation-wide shipping, which averages 19 h specifically for the overnight express option, incubation may be performed en route, allowing the incubation period to be fully completed during transportation and thus reducing the overall testing time. Our pathogen detection assays utilize a sandwich hybridization test for electrochemical detection of 16S rRNA content and currently include a 2 and 5-h viability culture for urine and blood, respectively^8^. Extended growth time often causes contaminants in urine, which would have otherwise remained below the clinical cutoff, to exceed the bacterial inoculum of the true infection. Therefore, the type of tubing as well as the thermal conditions of the packaging are both crucial to the regulation of such inaccuracies. Here, we demonstrate an evidence-based specimen transportation pack design (Fig. 1) considering the effects of weather conditions and transportation time on the growth of contaminants and bacteremia to further rapid direct-from-specimen antimicrobial susceptibility test (AST) and pre-hospital molecular phenotyping ID/AST diagnostics. The presented transportation protocol was developed as a first step towards performing AST directly from clinical specimens shipped from clinical settings. The differentiation of resistant pathogens from susceptible ones critically relies on the change of 16S rRNA content from viable pathogen after transportation, which is the main focus of this study and is tested with the pathogen detection assay only. A separate initial validation study focused on direct-from-specimen AST with categorical agreements of clinical specimens has been conducted (data not shown).

## Materials and methods

All procedures performed and samples collected in the presented study were in accordance with the ethical standards of the institutional and/or national research committee and with the 1964 Helsinki declaration and its later amendments or comparable ethical standards.

### Bacterial strains and assay materials

All samples tested were contrived with *E. coli* ATCC 25922, the only strain used in this study. All urine and blood specimens tested throughout the entire study were confirmed negatives, pooled, and tested on the day following collection. Specimens included de-identified remnant urine and blood (collected from CBC samples to ensure whole blood) collected from NYPQ for clinical diagnosis as part of standard care at NYPQ. This study did not involve any human subjects. Remnant clinical specimens were collected under a Non-Human Subject Research determination without consent (45 CFR 46 exemption 4) under the approved New York-Presbyterian/Queens Institutional Review Board and joint master agreement. The specimen volume used in the presented protocol was 2 mL for blood and 4 mL for urine, which is significantly lower than the typical collection volumes of 20 mL and 50 mL for blood and urine, respectively. Sensor chips used for the molecular detection of bacteria were produced in-house using a previously established protocol^8^. All bacterial counts for spiking were verified with blood agar plating.

### General shipping conditions

We optimized separate packaging conditions for urine and blood samples to comply with FedEx clinical shipping guidelines, which requires four layers of material: (1) primary watertight inner receptacle, (2) absorbent material, (3) secondary watertight inner receptacle, and (4) sturdy outer packaging. Urine samples were prepared in BD C&S preservative tubes (BD364951; Becton, Dickinson and Company, Franklin Lakes, NJ). Blood samples were prepared in tubes with no additive (BD366703) with specimen collected in tubes containing lithium heparin (BD367880). Tubes were then taped shut for an additional seal, wrapped in an absorbent material such as paper towel or cloth padding, and placed inside a sealed plastic bag. For urine samples, this bag was placed inside a plastic box with one cold pack. For blood samples, this bag was placed in an additional thermal bag with two heat packs wrapped in bubble wrap, reaching up to 40 °C, and was then placed inside the outer plastic box. A traceable thermometer was also included in each package to track the temperature profile of shipped specimens.

### Evaluation of urine shipping conditions

The initial effort to evaluate urine storage conditions for microbiological cultures was to compare the detection sensitivity of contrived urine samples at concentrations below and above the clinical cutoff of 10^5^ CFU/mL to mimic skin flora contaminations and uropathogens, respectively. We used a limit of blank (LoB) of 50 nA as the cutoff for reporting viable pathogen after transportation in all experiments. Results exceeding this cutoff indicated detection of viable bacteria; results below the cutoff were considered not detected. Because transportation times may vary depending on distance and other unpredictable factors, we tested the longest possible time, or worst possible scenario, of 3 days to simulate a shipment taking place over the weekend using various shipping and assay conditions. To first assess the effects of storage temperature, we prepared multiple sets of 4-mL urine samples in boric acid tubes, each containing two samples spiked with 1 × 10^3^ and 1 × 10^5^ CFU/mL *E. coli*, and tested them on Day 0 and Day 3 using three conditions. The two sets of samples tested on Day 0 did not undergo any storage conditions as they were tested immediately after sample preparation with either 1 or 2 h of viability culture in Mueller–Hinton II broth (Teknova; Hollister, CA) at 37 °C. In the first condition, one set of samples was stored at 4 °C for 3 days and allowed 1 h to return to room temperature just before testing with a 1-h viability culture. In the second condition, two sets of samples were stored at 4 °C and tested with either 1 or 2 h of viability culture immediately at the end of the 3-day period, with no additional hour to return to room temperature prior to testing. The third condition was similar to the second condition with the exception of room temperature storage rather than 4 °C storage.

As most clinical specimens are transported in 24 h or less, we wanted to evaluate the detection sensitivity throughout this more clinically relevant time period, rather than the 3-day period used in the previous experiment, by testing at 0, 12, and 24 h (lower and upper limits of overnight shipping time). Urine samples were prepared with *E. coli* at 1 × 10^2^, 1 × 10^3^, 1 × 10^4^, and 1 × 10^5^ CFU/mL. Two shipping and assay conditions were tested during this experiment. One condition included samples prepared in boric acid tubes and packaged with one cold pack that were tested with either 1 or 2 h of viability culture. The second condition was designed to evaluate the use of tubes without any additive and with one heat pack, essentially taking no additional measures to preserve the bacterial sample. Samples were then tested at 0, 12, and 24 h with a manual viable pathogen detection assay.

After various efforts to optimize shipping conditions, we tested an enhanced shipping protocol by simulating an overnight shipment in-house. We prepared two sets of urine samples per condition in boric acid tubes, with each set including one negative urine sample (“blank”) and samples spiked with *E. coli* at 5.5 × 10^2^, 5.5 × 10^3^, and 5.5 × 10^4^ CFU/mL. The first set (Day 0, before shipping) was tested immediately after preparation with 1 and 2 h of viability culture. The second set (Day 1, after shipping) was packaged with one cold pack according to the optimized shipping protocol and tested the following day after the simulated “overnight shipping” period in the warehouse, or the 19 h from sample preparation to an 8AM morning delivery, with a 1 and 2-h viability culture.

A summary of conditions for all three urine experiments can be seen below in Table 1.

**Table 1:** Summary of conditions for urine experiments.

Experiment 1—Test 10^3^ and 10^5^ CFU/mL in C&S preservative tube				
Condition	Day of test	Storage	Time to return to RT after storage/before testing	Assay viability culture
N/A	Day 0	N/A	N/A	1 and 2 h
1	Day 3	4 °C	1 h	1 h
2	Day 3	4 °C	0 h	1 and 2 h
3	Day 3	RT	N/A	1 and 2 h

Experiment 2—Test 10^2^, 10^3^, 10^4^, and 10^5^ CFU/mL at 0, 12, 24 h				
Condition	Tube	Storage	Assay viability culture	
1	C&S Preservative (boric acid)	1 cold pack	1 and 2 h	
2	No additive	1 heat pack	0 h	

Experiment 3—Overnight shipping of blank, 5.5 × 10^2^, 5.5 × 10^3^, and 5.5 × 10^4^ CFU/mL				
Condition	Hour of test	Storage	Assay viability culture	
N/A	0	N/A	1 and 2 h	
1	19	1 cold pack	1 and 2 h	

### Evaluation of blood shipping conditions

The main consideration for the design of the blood transportation pack was to enhance the recovery rate of viable bacteria, especially for an extremely low colony count (< 1 CFU/mL). Because lower colony counts require longer culture times to reach the limit of detection, we assessed the recovery rate of varying concentrations of bacteria in blood by testing 2-mL samples prepared in no additive tubes with *E. coli* at concentrations of 0.47, 4.7, and 47 CFU/mL. All samples were packaged in thermal bags with 2 heat packs and tested every 2 h for 10 h with the blood pathogen detection assay. The contrived density was verified by blood agar plating.

We then wanted to assess the described conditions under transportation times longer than the 10 h tested previously and observe if this longer transportation would lead to overgrowth of bacteria and affect detection sensitivity. Additionally, we wanted to further optimize the incubation conditions during transportation by testing the number of heat packs. To simulate overnight shipping, we prepared blood samples spiked with *E. coli* at 0.83 and 5.3 CFU/mL with a 2-mL starting volume. Samples underwent red blood cell lysing, followed by resuspension in Mueller–Hinton II (MH) broth, and were packaged with either one or two heat packs to simulate the overnight transportation time of 15–20 h before testing. This incubation period served to replace the viability culture portion of our blood pathogen detection assay. Samples were then tested with the blood pathogen detection assay upon receipt.

### Assessment of feasibility of transportation protocols

To test our improved preparation and shipping protocols for both urine and blood specimens, we asked New York-Presbyterian Queens Hospital (NYPQ) to contrive and ship urine and blood samples using optimized conditions. Urine samples were prepared in C&S tubes at 1 × 10^3^, 1 × 10^4^, and 1 × 10^5^ CFU/mL *E. coli* and packaged with one cold pack. Since there is a clinical cutoff for urine pathogen ID, a cold pack and boric acid were used to inhibit the growth during transportation. Blood samples were prepared in tubes containing no additive at 1 and 5 CFU/mL *E. coli* and packaged with no heat packs due to a shortage at the time of testing. Samples were packaged according to FedEx guidelines and shipped overnight to GeneFluidics. After approximately 22 h from the time of sample preparation to the time of delivery, all samples were tested with their respective pathogen detection assays.

### Viable pathogen detection assay procedure

Urine samples of 4-mL starting volume were spun in a centrifuge at 5000 RPM for 5 min, after which supernatant was removed and replaced with cation-adjusted MH broth. Samples were then cultured according to the conditions of the experiment. After the viability culture, the samples underwent a second round of centrifugation and supernatant removal, leaving 150 μL of sample. The samples were then lysed by adding 1 M NaOH with a 5-min incubation at room temperature, followed by the addition of 1 M HCl. Lysate was then delivered to all sensors on the sensor chip, with no sample being delivered to the negative control sensor. The chip was incubated for 30 min at 43 °C, then washed with distilled water to remove non-specific binding and dried with pressurized air. Horseradish peroxidase was delivered to every sensor on the chip and incubated for 5 min before a second wash and dry cycle. TMB was subsequently added to each sensor on the chip. After a 30-s incubation, the electrochemical signal generated by the chemical reaction of the HRP with the TMB and the applied voltage across the gold electrodes was measured by a 16-channel potentiostat reader.

Blood samples starting at 2 mL underwent a similar assay procedure with the exception of two additional rounds of red blood cell lysing before the addition of MH broth. Prior to packaging and culturing in MH broth, samples were lysed two times with 100 mg/dL saponin and incubated at room temperature for 10 min, followed by 5 min of centrifugation. Blood samples did not undergo a viability culture during the assay, as they were cultured during transportation according to the conditions of each experiment.

## Results

### Urine storage conditions to maintain detection sensitivity

**Figure 2:**
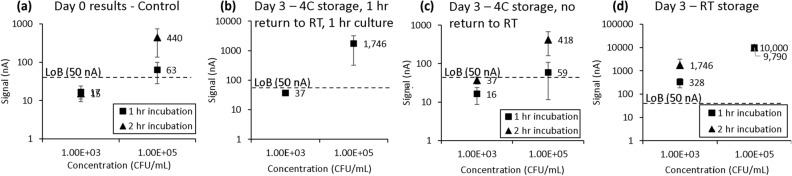
(**a**) Day 0 and (**b**–**d**) Day 3 viable pathogen detection assay results for contrived *E. coli* urine samples, comparing different storage and assay conditions. All samples were stored in C&S tubes. (**b**) Condition 1: 4 °C storage over 3-day period; given 1 h to return to RT prior to assay start time; assay included a 1-h viability culture. (**c**) Condition 2: 4 °C storage over 3-day period, no time given to return to RT prior to assay; assay included a 1 or 2-h viability culture. (**d**) Condition 3: RT storage over 3-day period; no time given to return to RT prior to assay; assay included a 1 or 2-h viability culture. Each error bar represents 7–8 data points; limit of blank (LoB) shown for reference.

The desirable conditions should report both contamination and uropathogens after storage and transportation the same way as tested immediately at T = 0 (Fig. 2a). As seen in the test summary in Table 2, urine stored at 4 °C without an additional hour to return back to RT before testing (Fig. 2c) resulted in pathogen detection reporting and signal levels similar to those generated during immediate testing (Fig. 2a). Samples of 10^5^ CFU/mL, which are at the clinical cutoff of 10^5^ CFU/mL, remained positive (above the limit of blank); samples of 10^3^ CFU/mL, which were reflective of skin flora contamination, remained negative (below the limit of blank). Much higher signal levels for both concentrations, but more importantly for 10^3^ CFU/mL samples, were observed in Fig. 2b,d, indicating bacterial overgrowth, which could result in false positives caused by overgrown contaminants.

**Table 2:** Test conditions and pathogen detection result summary from Fig. 2.

Conditions	Contrived urine at 10^3^ CFU/mL	Contrived urine at 10^5^ CFU/mL
Control set tested immediately at T = 0	Not detected with either 1 h or 2 h viability culture	Reported positive with both 1 h and 2 h viability culture
Store at 4 °C with 1 h return to RT (1b)	Not detected with 1 h viability culture	Reported positive with 1 h viability culture with exceedingly high signal indicating overgrowth
Store at 4 °C without return to RT (1c)	Not detected with either 1 h or 2 h viability culture	Reported positive with both 1 h and 2 h viability culture
Store at RT (1d)	Not detected with 1 h viability culture, but reported positive with 2 h viability culture	Reported positive with both 1 h and 2 h viability culture with exceedingly high signal indicating overgrowth

**Figure 3:**
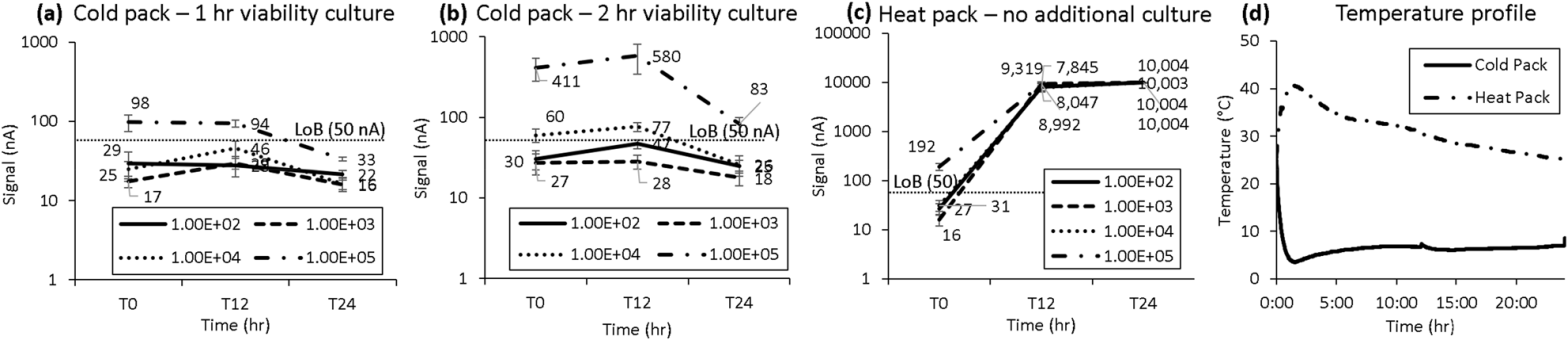
Urine calibration curve tested at 0, 12, and 24 h. Urine stored with one cold pack and tested with either (**a**) one or (**b**) 2 h of viability culture. (**c**) Urine stored with one heat pack and cultured for 1 h. (**d**) Thermal tracking profile of both transportation packages, including either cold or heat packs, used for experiment. Each error bar represents 6–15 data points; limit of blank (LoB) shown for reference.

In a follow-up experiment to test a more clinically relevant timeframe, we found that for urine samples packaged with one cold pack and tested with only 1 h of viability culture, those below clinical cutoff (10^2^, 10^3^, 10^4^ CFU/mL) were reported negative while urine samples contrived at 10^5^ CFU/mL were reported positive after 12 h of transportation. However, after 24 h of transportation the 10^5^ CFU/mL sample was reported negative, indicating that a longer viability culture is needed to bring the pathogen out from the stationary phase. A 1-h and 2-h viability culture were both tested, as shown in Fig. 3a,b, respectively, and we found that the 10^5^ CFU/mL sample was reported positive and exceeded the limit of blank after 12 and 24 h of transportation with a 2-h viability culture. For this condition, signal levels from T12 (12-h transportation) were comparable to those from T0 (tested immediately). If the specimen is transported through a commercial carrier such as FedEx, the temperature inside the delivery truck could be highly elevated as simulated with a heat pack in Fig. 3c, causing overgrowth of all contrived conditions. Figure 3d shows the thermal profile recorded with a traceable thermometer inside the transportation pack with cold or heat packs.

### Simulated urine specimen transportation

**Figure 4:**
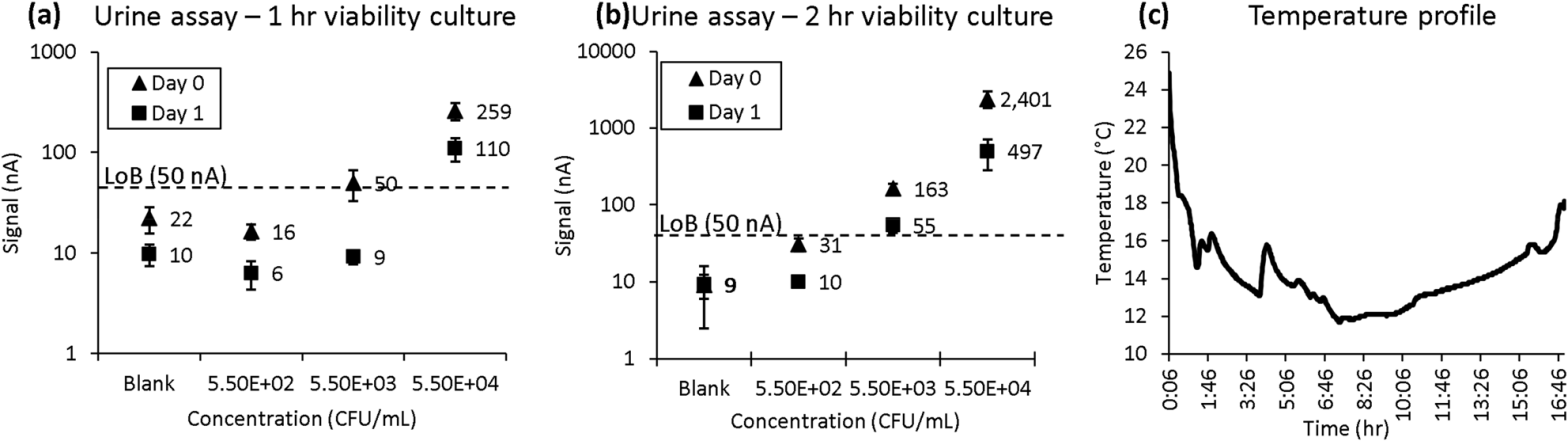
Simulated urine specimen shipment using the transportation pack finalized from Figs. 1 and 2. Calibration curves of urine assays including (**a**) 1 or (**b**) 2 h of viability culture. (**c**) Thermal tracking profile for transportation package used for experiment. Each error bar represents 8–15 data points; limit of blank (LoB) shown for reference.

Results for a simulation of overnight shipping of urine specimens performed in the warehouse showed that detection sensitivity was comparable between Day 0 and Day 1 if the pathogen detection assay included a 2-h viability culture. The urine sample spiked at 5.5 × 10^3^ CFU/mL could not be detected after FedEx Clinical Pak shipping if the viability culture was only 1 h as shown in Fig. 4, again confirming the need for a longer viability culture to bring the bacteria out of the stationary phase. However, these results show concentrations below the clinical cutoff exceeding the limit of blank for this detection assay.

### Blood specimen transportation pack to enhance viable recovery rate

**Figure 5:**
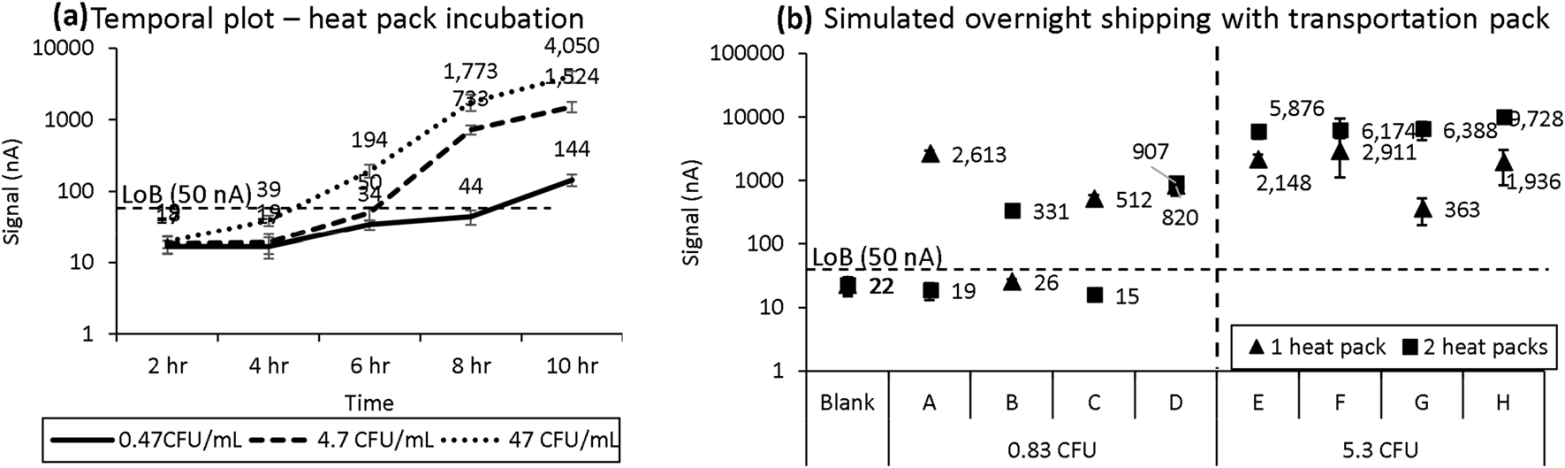
Simulated enhanced viability recovery of contrived blood samples at 0.47, 4.7, and 47 CFU/mL tested every 2 h after storage in a thermal bag with 2 heat packs. (**a**) All blood samples reported positive after 10 h of simulated transportation time. (**b**) Simulated overnight shipping with blood samples contrived at 0.83 and 5.3 CFU/mL with either 1 or 2 heat packs. Each error bar represents 5–15 data points; limit of blank (LoB) shown for reference.

Results from an effort to enhance the viable recovery rate of blood samples confirmed that low colony samples would require a longer viability culture time to reach the limit of detection to be reported positive (above 50 nA). In Fig. 5a, contrived blood samples reported positive after 6 h for 4.7 and 47 CFU/mL and 10 h for 0.47 CFU/mL. Table 3 shows the blood agar plating to verify the contrived concentration. In a simulated overnight blood specimen transportation via FedEx Clinical Pak shipment with contrived whole blood at 0.83 and 5.3 CFU/mL, two heat packs were needed to report both concentrations positive as shown in Fig. 5b. All four samples spiked at 5.3 CFU/mL produced positive results with one or two heat packs, although those packaged with one heat pack generated a lower signal level than those packaged with two heat packs, likely due to a lower average temperature profile. For the set of samples spiked at 0.83 CFU/mL, there was at least one sample for each heat pack condition that produced a negative result, as expected due to the probability of one colony existing in the 2-mL blood volume at 0.83 CFU/mL.

**Table 3:** Colony count at each time point of testing for Fig. 5a.

Concentration (CFU/mL)	2 h	4 h	6 h	8 h	10 h
0.47	0	65	500	Too many to count	Too many to count
4.7	11	199	Too many to count	Too many to count	Too many to count
47	164	500	Too many to count	Too many to count	Too many to count

**Figure 6:**
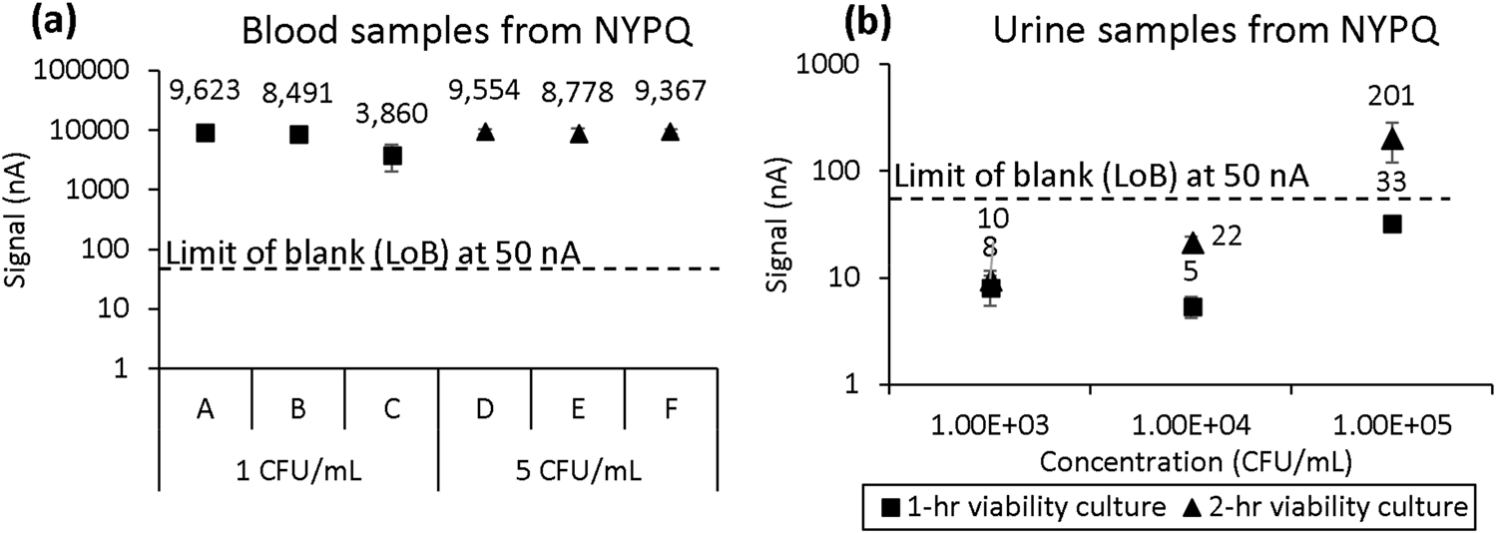
Feasibility study of specimen transportation of contrived (**a**) blood and (**b**) urine samples shipped from NYPQ to GeneFluidics through FedEx Clinical Pak overnight express. Each error bar represents 5 data points.

An additional experiment in which NYPQ prepared and shipped blood and urine specimens overnight to GeneFluidics demonstrated the feasibility of our improved specimen transportation protocols for both blood and urine. As shown in Fig. 6a, all six contrived blood samples (three at 1 CFU/mL and three at 5 CFU/mL *E. coli*) were reported positive after approximately 22 h between sample preparation and testing following transportation. In Fig. 6b, only the *E. coli* urine sample contrived at 10^5^ CFU/mL and cultured for 2 h during the assay tested positive, and samples of concentrations reflective of potential skin flora contaminations (< 10^5^ CFU/mL) were not reported positive as designed.

## Discussion

Specimens submitted for microbiological testing require proper handling from the time of collection through all stages of transport, storage, and processing, but these three stages have never been utilized as part of a test to report pathogen identification results within 1 day. The patient-side specimen collection and transportation protocol can report contrived blood sample positive at concentrations as low as 0.83 CFU/mL upon receiving the FedEx Clinical Pak shipment from New York to Los Angeles. The urine collection and transportation protocol can report uropathogens contrived at > 10^4^ CFU/mL positive while reporting concentrations ≤ 10^4^ CFU/mL, common of contaminants, as negative. We identified the optimal conditions for urine specimens to include one cold pack in the transportation pack to preserve the bacteria during transportation and a 2-h viability culture during the assay to bring the bacteria out of the stationary phase. For blood specimens, the ideal conditions included two heat packs, instead of one, wrapped in bubble wrap in the transportation pack to allow the samples to experience a higher average temperature throughout shipment.

The protocol optimization goals are different for whole blood and urine. We added heat packs to the blood transportation pack to keep bloodborne pathogens in the log phase and enhance the viability growth during the transportation period. We used cold packs for urine transportation to avoid bacterial overgrowth causing false positives with skin flora contamination. The number of cold or heat packs added to the package can change the thermal profile, and we identified an optimal condition for both blood and urine transportation packaging. The presented transportation protocol takes into consideration the clinical and technical factors that may have an impact on collection and transportation as detailed in other studies^9,10^.

To avoid false positives from skin flora, boric acid is routinely used to preserve the viable colonies. However, we observed a drop in signal level from our molecular analysis assay quantifying the 16S rRNA content of viable pathogens. The presence of boric acid and long transportation time could put the uropathogens into stationary mode resulting in lower RNA content caused by decreased colony count over time. We addressed this issue by adding viability culture time as part of our automated molecular analysis assays as shown in Fig. 4b. The closed-loop controlled thermal profile inside the system provided a consistent environment to bring the uropathogens back to log phase. The detection sensitivity was set to meet the clinical cutoff of 10^5^ CFU/mL, and the total assay time can be adjusted to achieve different levels of limit of detection by varying the viability culture time inside the system.

The main focus of the blood collection and transportation protocol development falls on the ability to detect low abundancy (< 1 CFU/mL) of pathogens in whole blood samples. Therefore, the primary goal is to prevent the loss of pathogen due to extreme conditions, and the secondary goal is to enhance the viable colony count during transportation. Figure 5a suggests that the transportation time needs to be longer than 6 h in order to call 5 and 50 CFU/mL positive, and 10 h for 0.5 CFU/mL. If the actual transportation time is less than the required time, the total assay time with the automated system at the receiving end will be adjusted based on the sample scan time stamp. No false positives were observed from all negative blood samples. The current blood specimen processing protocol is part of the automated procedure done by the robotic system. A separate specimen processing unit needs to be implemented at the patient site in order to automate this procedure to avoid specimen contamination and reduce the burden of healthcare professionals. Our aim was to utilize the transportation time as the viability culture time to achieve these goals; therefore, we included heat packs to simulate the conditions typically experienced during viability culture. There may be concern about potentially culturing contaminants during this period in addition to target pathogens, leading to false positive results. While this concern is valid and may even be shared with current blood culture methods, there are multiple factors that will need to be taken into consideration on a case-by-case basis to differentiate target pathogens from contaminants: collection site, species, and ratio of molecular quantification of target to contaminant^1^.

The pilot feasibility transportation testing is shown in Fig. 6. NYPQ staff at the clinical microbiology lab followed the work instruction to contrive, pack, and ship urine and blood samples through FedEx Clinical Pak to GeneFluidics. All samples were tested upon receiving, and the blood sample results agreed with the simulated study shown in Fig. 5b; all contrived blood samples generated signal above the limit of blank. For the urine sample results, there was disagreement between Figs. 6b and 4 results. Specifically, Fig. 6b showed ideal results, in which samples below the clinical cutoff of 10^5^ CFU/mL were below the limit of blank while the sample at the clinical cutoff was above the limit of blank. However, in Fig. 4, samples below the clinical cutoff were above the limit of blank, likely due to the simulated shipping in this figure being performed in a warehouse, which does not accurately reflect the environmental conditions and impact on samples experienced in an actual cross-country shipment, as mentioned in other studies^11^. Considering the variability of the ambient temperature inside delivery trucks, further validation studies are still needed to assess the impact of the thermal profile inside the specimen package by the outside environment.

Additionally, one limitation of this study was the use of only one bacterial strain for contriving samples. Urine and blood samples often present a variety of species generally observed in each specimen type, and these different species could display different phenotypic characteristics during transportation. Therefore, a larger panel of species and sample size should be tested to evaluate the feasibility of this protocol.

Timely tracking of infections and other causative-pathogen-specific events can facilitate antibiotic stewardship program quality improvement and monitoring of the effectiveness of infection management measures^12–14^. The presented protocol demonstrated the detection of viable pathogen after transportation and serves as an initial step toward leveraging the outcome of the current molecular analysis platform and establishing a patient-side initiated diagnostics platform to assess multivariable factors such as species and phenotypes of causative pathogens and microbiological response to antibiotics. Further clinical research is needed to see how these results relate to clinical measurements of success in combination therapies for sepsis in nursing home and pre-hospital settings. Success in such treatments is the consequence of a multitude of factors, including pharmacokinetics, effective in vivo drug concentrations, microbial species and host interactions. The outcome of this study could aid to conduct a future longitudinal study on the correlation of sepsis combination therapy efficacy and the emergence of AMR by providing timely reporting of causative pathogen and susceptibility profile and ability to monitor the changes in microbial load or susceptibility during antimicrobial therapy of outpatients.
